# Supplementary material for: A Novel Bayesian Framework Infers Driver Activation States and Reveals Pathway-Oriented Molecular Subtypes in Head and Neck Cancer
Source: Cancers (Basel). 2022 Oct 3;14(19):4825. doi: 10.3390/cancers14194825 (PMC9563147; doi:10.3390/cancers14194825)
Supplement: Supplementary file 1 [file cancers-14-04825-s001.zip › SupplementaryFIgureLegends.pdf]

Supplementary Figures

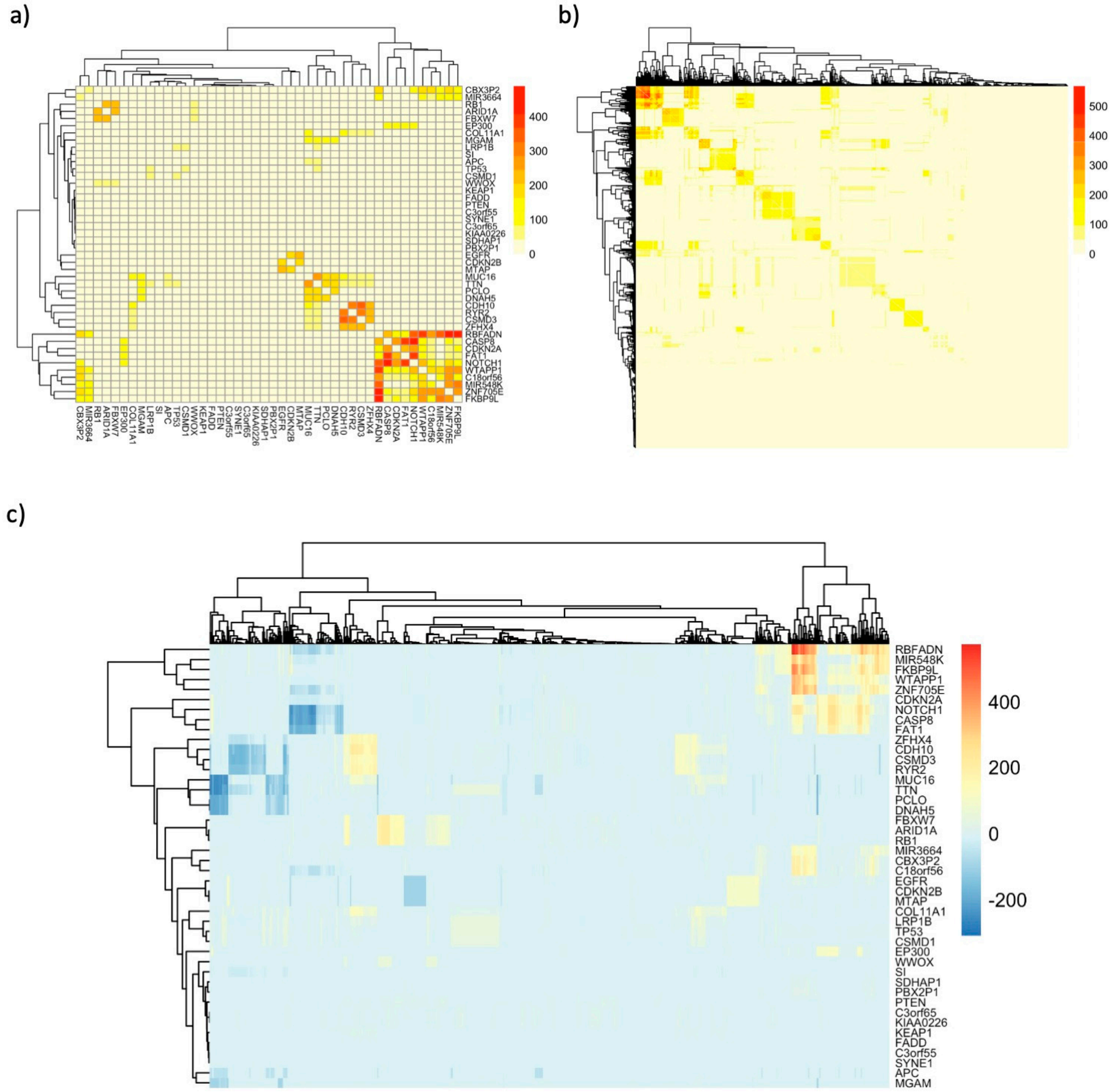

Supplementary Figure S1. Consensus clustering of drivers and DEGs. a) Consensus clustering of 64 TCI drivers based on number of times two drivers coregulating the same set of DEGs. b) Consensus clustering of 903 TCI DEGs based on number of times two DEGs regulated by the same set of drivers. c) Heatmap for identification of driver and DEG bi-clusters.

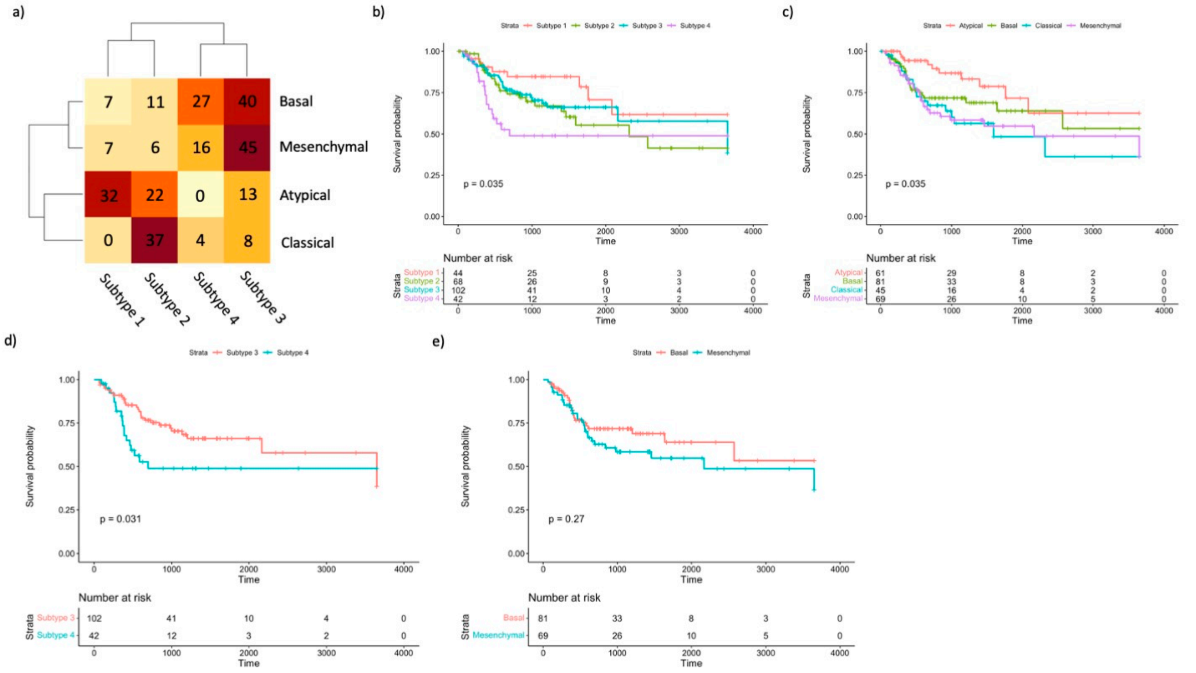

Supplementary Figure S2. HNSCC patient subtype comparison between our study and previous TCGA molecular subtyping. a) Heatmap of patient overlaps between our subtypes and TCGA molecular subtypes. b) Survival analysis of patients in our four HNSCC subtypes. c) Survival analysis of patients in TCGA HNSCC subtypes. d) Survival analysis of patients in our subtype 3 and subtype 4 which primarily comprise of a mixture of TCGA HNSCC Basal subtype and Mesenchymal subtype. e) Survival analysis of patients in TCGA HNSCC Basal subtype and Mesenchymal subtype.

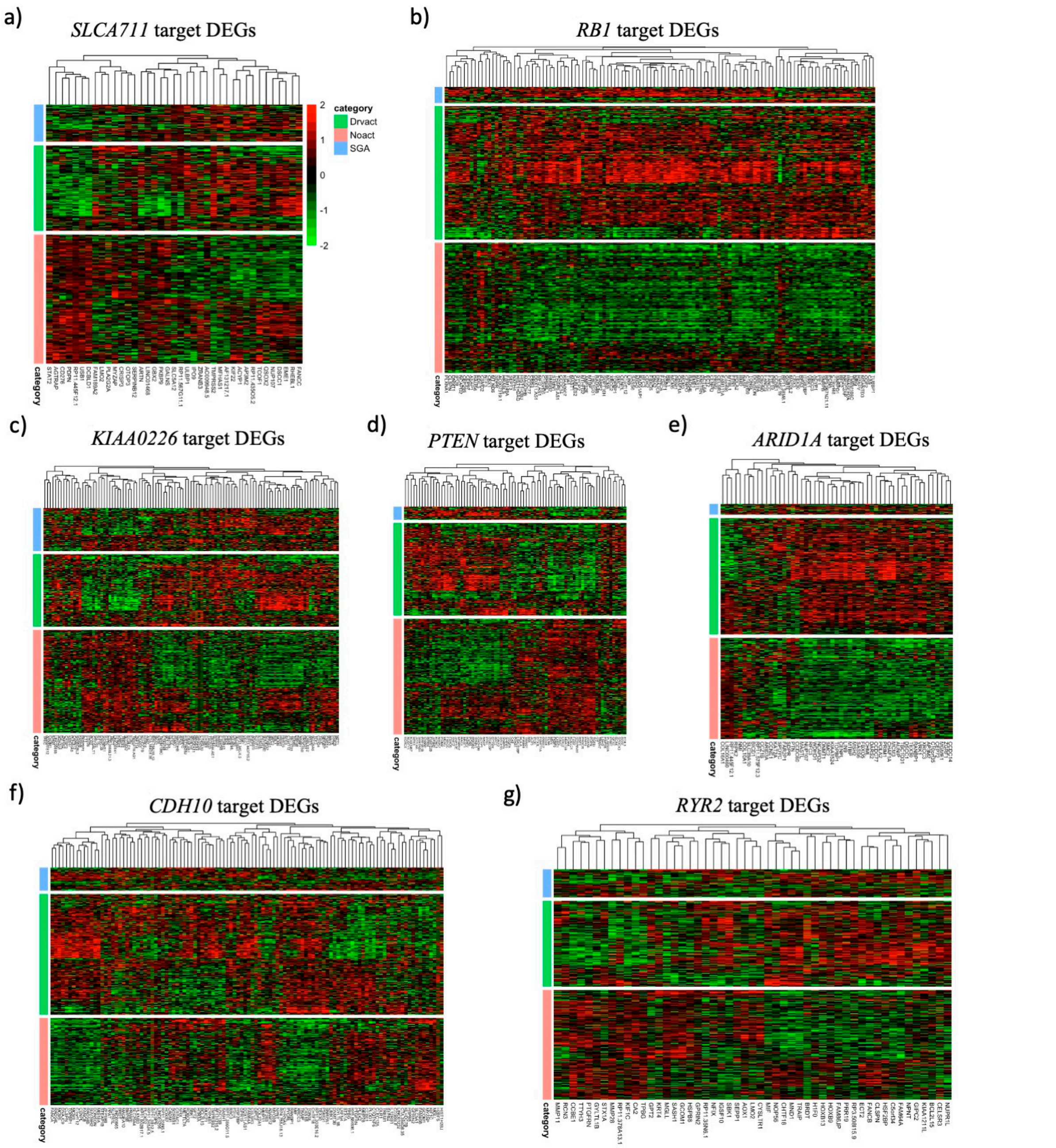

Supplementary Figure S3. Expression profiles of TCI-derived driver targeted DEGs in three groups of patients, i.e., patients with driver SGA, patients with inferred driver inferred protein activity and patients without driver SGA or inferred protein activity. These drivers have higher inferred protein activation rate but similar SGA frequencies in HPV-positive than in HPV-negative patients, including a) *SLCA711*, b) *RB1*, c) *KIAA0226*, d) *PTEN*, e) *ARID1A*, f) *CDH10* and g) *RYR2*.
